# Is bisphosphonate therapy for benign bone disease associated with impaired dental healing? A case-controlled study

**DOI:** 10.1186/1471-2474-12-71

**Published:** 2011-04-10

**Authors:** Gelsomina L Borromeo, Caroline Brand, John G Clement, Michael McCullough, Wendy Thomson, Elly Flitzanis, John D Wark

**Affiliations:** 1 Melbourne Dental School, The University of Melbourne, 720 Swanston Street Victoria, 3010, Australia; 2 University of Melbourne, Department of Medicine, and Bone and Mineral Service Royal Melbourne Hospital, Parkville, 3050, Australia; 3 Clinical Epidemiology and Health Service Evaluation Unit, Royal Melbourne Hospital, Parkville, 3050, Australia

## Abstract

**Background:**

Bisphosphonates are common first line medications used for the management of benign bone disease. One of the most devastating complications associated with bisphosphonate use is osteonecrosis of the jaws which may be related to duration of exposure and hence cumulative dose, dental interventions, medical co-morbidities or in some circumstances with no identifiable aggravating factor. While jaw osteonecrosis is a devastating outcome which is currently difficult to manage, various forms of delayed dental healing may be a less dramatic and, therefore, poorly-recognised complications of bisphosphonate use for the treatment of osteoporosis. It is hypothesised that long-term (more than 1 year's duration) bisphosphonate use for the treatment of post-menopausal osteoporosis or other benign bone disease is associated with impaired dental healing.

**Methods/Design:**

A case-control study has been chosen to test the hypothesis as the outcome event rate is likely to be very low. A total of 54 cases will be recruited into the study following review of all dental files from oral and maxillofacial surgeons and special needs dentists in Victoria where potential cases of delayed dental healing will be identified. Potential cases will be presented to an independent case adjudication panel to determine if they are definitive delayed dental healing cases. Two hundred and fifteen controls (1:4 cases:controls), matched for age and visit window period, will be selected from those who have attended local community based referring dental practices. The primary outcome will be the incidence of delayed dental healing that occurs either spontaneously or following dental treatment such as extractions, implant placement, or denture use.

**Discussion:**

This study is the largest case-controlled study assessing the link between bisphosphonate use and delayed dental healing in Australia. It will provide invaluable data on the potential link between bisphosphonate use and osteonecrosis of the jaws.

## Background

Bisphosphonates are non-metabolised analogues of pyrophosphates that are often prescribed to treat osteoporosis, Paget's disease of bone, metastatic osteolytic lesions associated with breast cancer, multiple myeloma, severe forms of osteogenesis imperfecta and moderate to severe hypercalcemia associated with malignancies [1-4]. In recent times intravenous bisphosphonates have also been advocated for the management of osteoporosis [5-7].

Post-menopausal osteoporosis is a common condition [8]. Less potent bisphosphonates such as alendronate and risedronate are first-line therapy for the treatment of post-menopausal osteoporosis, especially following a minimal-trauma fracture [9-11]. Paget's disease is another relatively common benign bone disorder for which bisphosphonates are first-line treatment [12]. By 2006, over 19 million prescriptions for bisphosphonates were dispensed worldwide suggestive of a good safety profile and benefit in osteoporosis management [13].

Adverse events related to bisphosphonate usage have centred largely around gastrointestinal and renal safety, bone, joint or muscle pain and the development of acute phase reactions [14]. Several recent reports have highlighted the possible association between bisphosphonate use, especially intravenous (IV) zoledronate or pamidronate, and jaw osteonecrosis [15-21]. Other studies have not found any association between bishosphonate use and ONJ [6]. A study assessing once yearly zoledronate for osteoporosis management in 8000 individuals reported, following separate case adjudication, a single episode of ONJ in each of the placebo and zoledronic acid groups [6]. The length of this study was only three years whereas most ONJ has been reported in patients taking bisphosphonates for longer periods of time. In a recent survey of over 8000 respondents, ONJ had a prevalence of 0.10% (95% confidence interval 0.05% to 2.0%)[22]. Previously, similar but less frequent presentations with jaw osteonecrosis occurred following radiotherapy (Ruggerio et al., 2004) or occupational exposure to phosphorus [23]. In many of the reports, jaw osteonecrosis occurred in the setting of malignancy, in particular, multiple myeloma [24-26] or breast cancer [18,19,26]. Recent work has suggested that in myeloma patients, zoledronate and pamidronate are associated with a 10% and 3% incidence of jaw osteonecrosis at 36 months, respectively [25]. In contrast, the prevalence of jaw osteonecrosis with bisphosphonate treatment in a large retrospective case series was 2.4% among myeloma patients and 1.2% among breast cancer patients [26].

There is some published evidence that chronic low-dose bisphosphonate treatment for osteoporosis or other benign bone disease is associated with jaw osteonecrosis [18,27]. However, randomised controlled trials of bisphosphonates in osteoporosis have not demonstrated an increased risk of jaw osteonecrosis. The development of ONJ has been linked to duration of exposure to bisphosphonates, and hence a higher cumulative dose, longer duration of treatment, hence prolonged survival, as well as potential co-morbidities such as prednisolone or thalidomide use [26]. Poor periodontal status together with dental interventions, in particular extractions, implants or trauma from dentures for example, significantly increase the risk of developing ONJ in this patient cohort [17,19,20,26,28].

The exact mechanism by which bisphosphonates may contribute to impaired resistance to injury or impaired healing of the maxilla or mandible and to osteolytic destruction, remains unclear. However, suppression of bone turnover via actions on osteoclasts seems to play a substantial role [29,30]. Bisphosphonates are not the only medications with this action. Other drugs such as denosumab (a RANKL antibody which is indicated in cancer patients) and bevacizumab (a human monoclonal antibody to vascular endothelial growth factor) have the potential to alter osteoclast differentiation and function and as such has also been implicated in ONJ [31,32].

Although ONJ associated with bisphosphonate use appears to resemble osteoradionecrosis seen following jaw radiotherapy, they are now considered different disease entities [33]. Conventional therapy of this latter complication involves local debridement, irrigation and antibiotics [34]. However, this strategy has yielded mixed results in bisphosphonate-associated osteonecrosis, and may contribute to further tissue breakdown, resulting in large fistulas [35].

While jaw osteonecrosis is a devastating end-stage outcome that is currently difficult to manage, various forms of delayed dental healing may be a less clinically dramatic and, therefore, poorly-recognised complications of bisphosphonate use. Currently, the likelihood of dental complications during bisphosphonate therapy for treatment of post-menopausal osteoporosis or other benign bone disease is uncertain. It is unclear what factors predispose patients to these events [36,37]. Given the large numbers of patients receiving long-term bisphosphonate therapy, particularly in benign bone disease, that a high level of professional and public concern has arisen about the issue, and the fact that we are living in an ageing population making the likelihood of levels of osteoporosis increasing, understanding the prevalence and risk of bisphosphonates and jaw osteonecrosis is paramount.

## Hypothesis

The hypothesis to be tested is that long-term (more than 1 year's duration) bisphosphonate use for the treatment of post-menopausal osteoporosis or other benign bone disease is associated with impaired dental healing.

## Methods

### Study design

**Figure 1:**
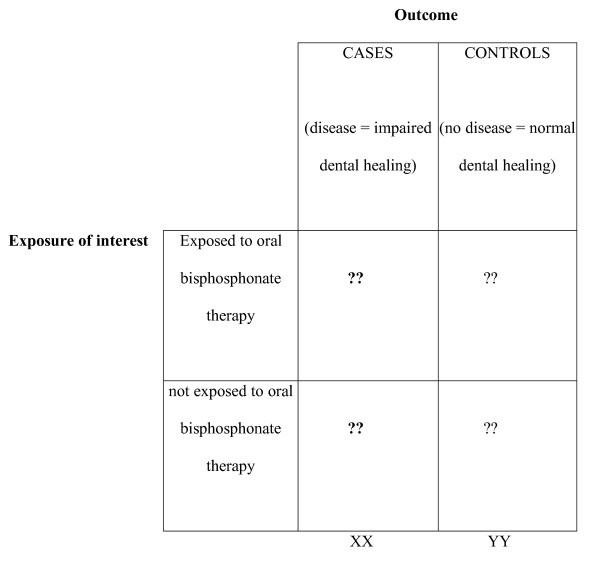
**Case-controlled study design**.

A case-control study has been chosen to test the hypothesis as the outcome event rate is likely to be very low (Figure 1).

### Definitions

#### Delayed dental healing

Delayed dental healing (a precursor to osteonecrosis of the jaw) is defined as a persistent breach in the oral mucosa and/or exposure of bone in the mandible or maxilla that:

• fails to heal within 6 weeks as documented by a dentist despite usual therapy;

• occurs either following a dental procedure, for example a tooth extraction or crown insertion, or spontaneously, with or without osteonecrosis.

#### Osteonecrosis of the jaw

• Exposed bone in the maxillofacial area that occurred in association with dental surgery or spontaneously with no evidence of healing

• No evidence of healing after 6 weeks of appropriate evaluation and dental care

• No evidence of the following bone pathology that might explain the findings: metastatic disease in the jaw or osteoradionecrosis.

### Setting and study time frames

**Figure 2:**
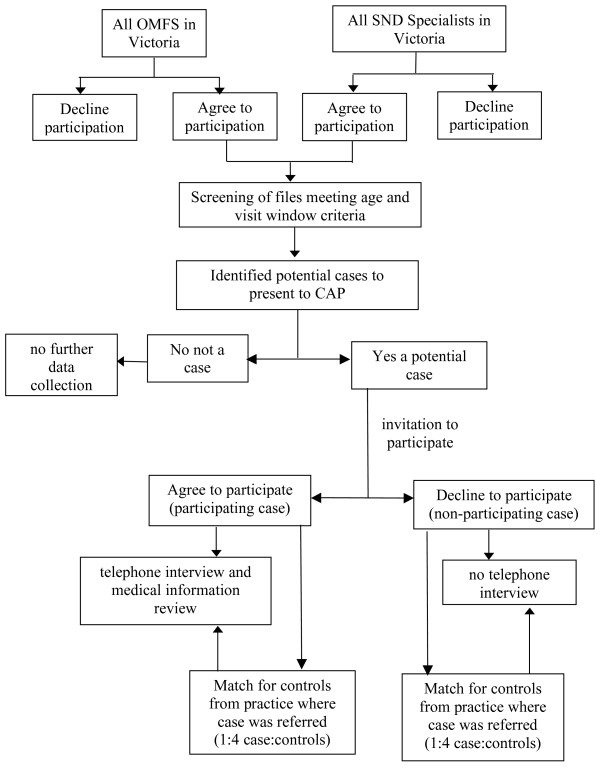
**Flow diagram of study protocol**.

The study will take place in Victoria, the second most populous State in Australia with a population of approximately 5 million people. The visit window period study period will be March 1^st ^2006 until August 31^st ^2006. Participants in the study will have been treated in specialist oral and maxillofacial and special needs dentistry settings during the visit window period. Control subjects will have attended local community based referring dental practices. A flow diagram of the study protocol is depicted in Figure 2.

### Dental Specialist Recruitment

Specialist dental recruitment will involve contacting all registered oral and maxillofacial surgeons and special needs dentists who were actively practicing during March 2006 through to the end of August 2006. All registered specialists listed in the Yellow Pages telephone directory will be cross-matched with those currently registered with the Australian Health Practitioner Regulation Agency. The researchers will also present the study to a Victorian Branch meeting of the Australian and New Zealand Association of Oral and Maxillofacial Surgeons. All public hospital dental specialty clinics in Victoria and associated specialist dental practitioners will be identified. All registered specialist dental practitioners will be invited to participate in the case-controlled study via introductory mail out.

Follow up of specialist non-respondents would occur by mail at 2-weeks and telephone at 4-weeks. Once specialists agree to participate, then the respective practice will be contacted to determine a suitable time to view patient files and identify potential cases. All files within the 6-month visit window period will be screened and only those meeting the age and visit window criteria will be reviewed for further potential case information. Once potential cases are determined then they will be presented to the Case Adjudication Panel (CAP) (see further details below).

### Case Recruitment

Recruitment of individuals for the study is a two-step process, ascertainment of potential cases through dental record review then verification by a case adjudication panel (CAP). The research team would visit each participating practice to identify potential cases from the dental records, which could then in turn be presented to the case adjudication panel. Each specialist practitioner would be required to give consent to have patients in their practice contacted by the research team.

Participant ascertainment will occur through consecutive screening of oral and maxillofacial and special needs dental specialist records in private practices and public hospitals by trained research staff. Once a case has been confirmed by CAP, each specialist practitioner would be required to give consent to have patients in their practice contacted by the research team.

To be eligible for participation, the dental record should indicate that the potential participant is:

• age ≥ 50 years

• has a dental wound that failed to heal within 6 weeks

• had a qualifying visit during the window period.

The exclusion criteria are:

• A history of active malignancy or malignancy within five years (excluding basal or squamous cell carcinoma).

• Previous radiotherapy field that included the jaws.

• Bisphosphonate use for any indication other than post-menopausal osteoporosis or other benign bone disease.

In order for potential cases to be identified from the patient files, the research team reviewing the clinical notes need to be able to identify delayed dental healing. It is expected that the term "delayed dental healing" as a potential descriptor is unlikely to appear in the clinical notes, as there would be variability in clinical descriptions amongst specialists. As such a number of file terminology descriptors will be used to help identify potential cases of DDH and these are outlined within 'data collection methods' below.

All records identified as potential cases of delayed dental healing will be presented to the CAP for verification for inclusion or exclusion. The CAP will include a Chair who will be an independent specialist endocrinologist, 2 other medical practitioners and three dentists (one Oral Medicine Specialist, one oral and maxillofacial surgeon and one forensic dentist/bone biologist). A quorum of 4 (2 dental and 2 medical) will be required at each CAP meeting.

At each CAP meeting all de-identified potential cases will be presented without the panel's knowledge of bisphosphonate history and medical status. The records presented will be assessed according to the previously stated inclusion and exclusion criteria.

Once verified as a definite case by the CAP, the individual will be contacted via mail to seek informed written consent for participation in the study. Non-responders will consist of two main groups; individuals who do not wish to participate and those who cannot be contacted (e.g. changed address or deceased).

### Recruitment of Control Subjects

Each case will be matched with four controls randomly selected from those who have undergone dental treatment at the same dental practice from which the case originated. If access cannot be obtained to records from the originating dental practice, then a similar type of practice e.g. private practice or hospital - based practice, within a 10 km radius will be approached to provide control subjects. They will be matched for age (>50 years), gender and visit window period and will have no known defect in dental wound healing. If controls cannot be matched to the 6-month visit window period then they will be matched to within 12 months of the visit window period. Controls will be contacted by mail as for cases to obtain informed consent to study participation.

### Data Collection Methods

Data collection will involve collecting information pertaining to demographics, delayed dental healing, bisphosphonate history and medical history. Data collection forms and telephone interviews for controls will be the same as that used for cases.

#### Part 1: Demographic information

Specialist information, patient details (name, date of birth, gender, address and telephone number), name of referring practitioner, referring practitioner contact details, date of presentation of oral problem and referral to specialist date will be collected initially. A list of all dates within the visit window period together with an outline of each visit will be recorded. The demographic data will be coded for each individual and the code used in all subsequent data collection in order to de-identify information as per ethical requirements.

#### Part two: Delayed dental healing information

Each patient file identified within the visit window period will be analysed and information relating to the presence of an oral diagnosis (diagnosis, site, precipitant, treatment, outcome, biopsy report, radiographic investigation), bisphosphonate history and medical history (co-morbidities and medications) will be recorded.

The diagnosis will consist of the presence of either oral ulceration ("break in mucosa but no bone visible") or bone necrosis defined as "bone on view". A number of key words will be used when reviewing patient histories as a number of descriptors can be used to describe delayed dental healing. These will include non-healing socket, pus, exudate, swelling, draining sinus, dry socket, bone sloughing, sore sockets, OAC, oroantral communication, healing not completed, fistula, OAF, oroantral fistula, exposed bone and infection.

Information regarding site of the lesion will be further subdivided into single versus multiple sites, quadrant involved, palatal, lingual, buccal or labial orientation and tooth area (one to eight). The main precipitants listed can include tooth extraction, implant insertion, removal of pathological lesion, denture use, spontaneous, no obvious precipitant or other. In each case the date of the precipitant will be recorded. If the precipitant is not recorded in the history this will be marked as such on the data collection form.

Outcome of the delayed dental healing will be recorded by including treatment modalities such as antibiotics, mouthwashes/irrigation, debridement or other, date of the last review together with wound status (healed completely, healed partially, no healing or not recorded) and progress of the wound at the last visit (worse, stable, improving or not recorded). Details regarding any biopsy or radiographic analysis will also recorded.

#### Part 3: Medical History

Detailed information relating to potential cancer history including type, date of diagnosis, remission status, radiotherapy to the jaw and chemotherapy will be recorded as this is a key exclusion criterion. Other co-morbidities will be recorded including lung disease, heart disease, kidney disease, organ transplant, diabetes, rheumatoid arthritis or other connective tissue conditions together with smoking, tobacco and alcohol intake as these may contribute to delayed dental healing.

#### Part 4: Medication history

A detailed description of oral glucocorticoids (start/stop dates, current dose and cumulative dose), hormone replacement therapy and other medications including raloxifene, calcitriol, tibolone, teriparatide and strontium will be recorded.

If a bisphosphonate has been prescribed, the type will be recorded including alendronate, risedronate, tiludronate, pamidronate, zoledronate or etidronate together with indication for use (osteoporosis, Paget's disease, glucorticoid-induced osteoporosis, metastatic disease, hypercalcemia). Doses of all bisphosphonates including start and stop dates and current doses will be recorded to allow calculation of cumulative drug doses.

#### Telephone interview

A telephone interview will be conducted with all consenting participants in order to confirm inclusion/exclusion criteria (most of which may already be evident from the patient's dental file) and to determine socio-economic status, dental health information and a medical history check including bisphosphonate history, medication profile and smoking history. The telephone interview will last approximately 10 minutes. The telephone questionnaire will be modelled on the Adult Oral Health Survey [38] and include questions relating to dental health and general information such as socio-economic status and educational status. A medication check list will also be completed with each case regardless of the data collected in the original data collection form in order to cross match bisphosphonate history, other medical history which could contribute to impaired wound healing and smoking history.

### Ethics Approval

Human Research and Ethics Committee approvals have been obtained from: Melbourne Health (2005.242) (hospital and private practice cases and controls), Austin Heath (H2006/02599; H2010/03794), The Alfred (17/09), Barwon Heath (10/99), Dental Health Services Victoria (197), Southern Health (09069A), St Vincent's Hospital (009/09) and Western Health (2005.242).

### Statistical Analysis

Demographic and clinical characteristics of cases and controls will be presented to assess whether these variables are associated with delayed dental healing.

#### Power and sample size

The sample size estimate is based upon the assumption that the overall prevalence of bisphosphonate use in post-menopausal women is around 10%. Based on Australian Pharmaceutical Benefits Scheme (PBS) and Australian Bureau of Statistics (ABS) data the upper limit estimate is 19% [39]. Assuming that approximately 50% of these patients have used bisphosphonate for at least one year, then 10% prevalence of long-term use in this population appears to be a reasonable estimate. It is hypothesised that the prevalence of bisphosphonate use among those with delayed dental healing may be greater than 10%. A recent case series of patients with frank jaw necrosis found that all were treated with bisphosphonates [19]. We infer from this that the true proportion of women with DDH taking bisphosphonates may be greater than 30% - 50%. Given the expected low prevalence of DDH, the study plans to recruit 'controls' and 'cases' in a ratio of 4:1. An observed prevalence of greater than or equal to 30% bisphosphonate use amongst women with DDH would correspond to an odds ratio of around 3.85. Hence, for the purpose of this study, the minimum detectable difference will correspond to an OR greater than or equal to 3.85. This value is based upon a conservative estimate of the effect expected, rather than what is considered clinically important. The most relevant measure of clinical importance will be one derived from a cohort study, that is where the effect of bisphosphonate use can be represented in terms of a relative and/or absolute risk of delayed dental healing. Whilst ideal, this would require a very large sample and long-term follow-up.

A total sample of around 269 subjects (54 cases and 215 controls) will provide 90% power to detect a true OR of at least 3.85, given the expected prevalence of 10% bisphosphonate use amongst post-menopausal women in the community.

The relationship between bisphosphonate use and delayed dental healing will be assessed using a multivariate logistic model incorporating age, duration of exposure, relevant co morbidities, concurrent treatment, and other potential confounders as covariates.

The prevalence of DDH in the target population will be estimated using data collected to determine case numbers in both bisphosphonate-treated and non-bisphosphonate-treated patients. We will also record cases of DDH occurring in non-bisphosphonate-treated patients with a diagnosis of benign bone disease where bisphosphonates may be indicated (i.e. osteoporosis, Paget's disease of the bone).

### Outcome measures

#### Primary outcome measures

The primary outcome will be the incidence of delayed dental healing that occurs either spontaneously or following dental treatment such as extractions, implant placement, or denture use.

#### Potential covariates

Potential covariates include those data collected, which increase our knowledge of the potential to develop DDH. These include co-morbidities (medication history, smoking history, other medical conditions), oral hygiene habits and demographics (socioeconomic status, nationality).

Bisphosphonate use is considered an explanatory variable, which is also our exposure of interest.

## Discussion

The present study seeks to determine the level of delayed dental healing that occurs either spontaneously or after dental procedures such as tooth extraction and how this relates to bisphosphonate usage. Delayed dental healing may be an earlier or less advanced lesion compared to ONJ but with a similar pathogenesis. By observing delayed dental healing as well as ONJ we may therefore more broadly describe bisphosphonate associated dental disorders and increase our power to find an association between bisphosphonate use and associated dental disorders. It is imperative to obtain a better understanding of this condition and its potential links to bisphosphonate use as it is often refractory to treatment and can lead to significant morbidity including bone sequestration, intraoral and extraoral fistula formation, secondary paraesthesia and pathological jaw fractures. The link between this and bisphosphonate use is paramount as there are a number of studies reporting the incidence of jaw osteonecrosis (a potential sequelae for delayed dental healing) to be as high as 0.09 - 0.34% in patients receiving oral and 6.7-9.1% in patients receiving intravenous bisphosphonates following dental procedures [40]. Furthermore, whilst the incidence of ONJ in osteoporosis patients in relation to bisphosphonate use has been reported to occur after prolonged treatment (greater than 3 years), it has been reported following 6-month use [41]. On the other hand, other data suggest an extremely low prevalence/incidence of ONJ in patients treated with bisphosphonates for osteoporosis and other metabolic bone disorders. Although an association between ONJ and bisphosphonate use has been suggested by case series, professional surveys and register data, there is a lack of controlled, population-based data. A key aim of the present study is to obtain such controlled data. By recording the prevalence of delayed dental healing and ONJ that occur in the absence of bisphosphonate use we hope to be able to estimate the true risk of these disorders in association with bisphosphonate exposure.

A case-controlled study design has been selected over a prospective cohort study. Whilst both are observational studies that could further knowledge of delayed dental healing, ONJ and bisphosphonate use, the former study design has a number of advantages. First, it will allow us to study an outcome with a potentially low incidence, less than 1% in patients with osteoporosis or Paget's disease [42]. Second, this approach will minimize the problem posed by a long latency between exposure to bisphosphonate therapy and the outcome of delayed healing and subsequent ONJ, something which cannot be accounted for easily in a prospective cohort study, except by extended follow-up. Third, we will be able to study the effects of other potential risk factors for delayed healing such as medical history including bisphosphonate usage, smoking history, dental hygiene, dental trauma including tooth extractions and implant placement, and denture usage on the outcome of interest, namely delayed dental healing. It is understood that a prospective cohort study would provide the most reliable assessment of the incidence of delayed healing, ONJ and bisphosphonates but to date such studies have not been conclusive [14].

One of the major limitations of this case-controlled study design is that it is reliant on information as it is recorded in the medical or dental history that may be incomplete and is subject to clinician bias and researcher interpretation. This is compounded by the fact that in 2006 there was considerable controversy regarding the role played by bisphosphonates in ONJ and the definition of the condition. Furthermore, resources required to access and collect data from thousands of medical and dental histories could result in a prolonged study period.

The present study also relies on recruitment of specialists to allow access to patient records followed by recruitment of cases and controls. All oral and maxillofacial specialists and special needs dentists in the state of Victoria will be invited to participate in the study but it will be difficult to control for ascertainment bias. Are practitioners who see delayed dental healing and ONJ more willing to allow review of their patient records? Are those who have a greater interest in the role played by bisphosphonates more likely to want to become involved? It is difficult to control for this even with the use of random sampling because practitioners' consent is required to allow file review. To some degree the same can also be said for patients with delayed dental healing. The patient information and consent brochure stipulates that this is an important study to further our understanding of the link between delayed dental healing and bisphosphonate use. Whether an individual who has taken a bisphosphonate may be more or less likely to participate is difficult to determine. Another potential limitation of the study is recollection bias given that some data will be collected via participant telephone interview. Furthermore, control subjects reading the patient information and consent brochure may feel that the study does not really benefit them and hence may be less likely to respond. The same can also be said for the general dental practitioners via whom the control subjects will be identified. If they have little experience with patients taking bisphosphonates or delayed dental healing, then they may have little motivation to allow a third party to access their patient records.

A key feature of the present study relates to the definition of DDH and ONJ. In 2005 the definition of osteonecrosis of the jaw was unclear and constantly changing in the literature and as such it was difficult to adhere to a single definition. The main disparity at the time was related to the length of time a wound took to heal before it fell into the category of osteonecrosis of the jaw. Initial healing, that is re-epithelialisation, of dental wounds such as those from dental extractions usually takes between 1 and 2 weeks [43]. Once the clot forms, fibrin and connective tissue begins to develop before the wound (in this case a dental socket) is closed over by epithelium. It then takes some weeks for the underlying socket to fill with bone and healing to be complete.

Hence up to 6 weeks for healing of a dental wound would be reasonable taking into account potential effects of delayed healing from medical co-morbidities such as steroid use or development and subsequent healing from complications such as a dry socket. Furthermore, in 2008, a report from the task force of the American Society for Bone and Mineral Research proposed that a "suspected" case of ONJ would be defined as "an area of exposed bone in the maxillofacial region that had been identified by a health care provider and have been present for less that 8 weeks" which was supported by others [42,44]. By this time, soft tissue closure and exposed bone would no longer be present. ONJ would then be the definitive diagnosis if greater than 8 weeks had lapsed for healing to occur [42,45,46]. The current definition for bisphosphonate associated ONJ includes the following features:

1. Current or previous treatment with a bisphosphonate

2. Exposed bone in the maxillofacial region that persisted for greater than 8 weeks and

3. No history of radiation therapy to the jaws [47].

An attempt also has been made to define clinical stages of ONJ [45,48] (AAOMS, 2007). Stage 1 involved the presence of exposed or necrotic bone that is asymptomatic with no evidence of infection. Stage 2 related to the presence of exposed necrotic bone and infection, erythema and the presence or absence of a purulent discharge. Finally stage 3, the most severe form of ONJ, contained all the characteristics of stage 2 but in addition, the presence of features such as a pathological fracture, draining sinus or communication either intra oral or extraoral and osteolysis. Since then, Stage 0 has been included to encompass patients with signs of ONJ but no exposed bone [46].

Potential covariates to be collected in this study are in line with those suggested as risk factors for ONJ and include dental factors such as tooth extraction, implant placement and denture use, treatment factors such as use of glucocortocoids and smoking status [26,29,49].

A critical component to the success or failure of any case-controlled study is recruitment of participants. The study was designed as a two-step recruitment process requiring not only patient participation but also clinician participation otherwise access to patient data and potential cases would have not been possible without employing more complex recruitment protocols. It is also imperative we seek the assistance of specialist practitioners in order to gain permission to screen consecutive patient records during the defined study time period in order to avoid selection bias associated with specialist recall of individual patient cases. Hence there are two potential problems with recruitment. The first lies with recruiting specialists. A number of key features have been identified to be essential to increase response rates in postal questionnaires [50]. A number of these features are also pertinent in the following study. Firstly, Edwards *et al.*, (2002) suggested that contacting participants before sending out questionnaires would be important. In the current study, the researchers presented the study outline and requirements of participants (clinician and patient) to oral and maxillofacial surgeons at a continuing professional development meeting. It is considered crucial to the study to recruit a high proportion of oral and maxillofacial surgeons as these are recognized as the group most likely to treat patients delayed dental healing following dental procedures. During this presentation the potential benefits of determining incidence of delayed dental healing with reference to bisphosphonate usage were discussed, which was considered to be of great interest to practicing specialists. This has also been determined as a potential method to increase response rate to postal surveys [50].

Other key factors employed to increase clinician recruitment included using personalized introductory letters, short questionnaires, follow-up letters to non-respondents and telephone follow-up. These are recognized strategies to increase recruitment [50]. Similar measures were also employed when recruiting potential cases and controls.

## Conclusions

The study uses a case-controlled design to assess the hypothesis that long-term (more than 1 year's duration) bisphosphonate use for the treatment of postmenopausal osteoporosis or other benign bone disease is associated with impaired dental healing and subsequent development of ONJ. All Victorian Oral and Maxillofacial Surgeons and Special Needs Dentists, by far the largest groups managing these patients, will be invited to participate making this the largest such study in Australia.

## Competing interests

The authors declare that they have no competing interests.

## Authors' contributions

GLB conceived the project and has been involved in co-ordination of the project. GLB, CB, JC, MM and JDW assisted with protocol design and obtaining funding. GLB, WT, EF and MM are involved in data collection. GLB, CB and JDW wrote the manuscript. All authors provided feedback on drafts of this paper and read and approved the final draft before submission.

## Authors' information

GLB is a Special Needs Dentist, senior lecturer and researcher, JC is a Bone Biologist and Forensic Dentist and MM is an Oral Medicine Clinical Specialist and Academic Associate Professor. CB is a consultant Physician, Rheumatologist and Health Services Researcher and JDW is an Endocrinologist and Professor of Medicine with major interest in bone and mineral disorders. WT and EF are research assistants.

## Pre-publication history

The pre-publication history for this paper can be accessed here:

http://www.biomedcentral.com/1471-2474/12/71/prepub
